# QOI9/476: Online Prescribing of Sildanefil (Viagra) on the World-Wide-Web

**DOI:** 10.2196/jmir.1.suppl1.e102

**Published:** 1999-09-19

**Authors:** G Eysenbach, TL Diepgen

**Affiliations:** ^1^Institute for Clinical Social Medicine, HeidelbergGermany

**Keywords:** Consumer-Physician Interaction, Remote Consultation, Quality of Health Care, Online-Prescription, Viagra (Sildanefil)

## Abstract

**Introduction:**

The World-Wide-Web has become a medium to advertise and dispense medicines directly to consumers. Little is known about the structure and "quality" of these "virtual pharmacies" in terms of how responsible "online-prescriptions" are actually issued.

**Methods:**

We simulated a patient in which the ordered drug (Viagra) is clearly contraindicated and tried to purchase this drug on the Internet. Our test case was as a 69 year old woman giving a sexual history of having "no orgasm", with obesity (165cm/78kg), coronary artery disease and hypertension, taking captopril, pravachol, atenolol and erythromycin.

**Results:**

22 distinct companies were identified, consisting of three different types: 2 required a written prescription by a "real" physician, 9 dispensed the drug without any prescription at all, and 11 issued an "online prescription" after the an alleged physician reviewed the online order form containing medical questions. We tested 10 of the latter type, among them 8 based in the USA. We ordered a total of 66 pills worth 1.802,84 US$. 3 companies, among them both Europeans, delivered within 6, 10 and 34 days respectively, despite Viagra being clearly contraindicated. In 80% no complete history was taken, in 70% inappropriate medical terminology was used, and in only two cases the order form was reviewed by a physician who identified himself.

**Discussion:**

Although a surprisingly high number of Internet pharmacies declined delivery, the public should be alerted about the risks involved with prescription drug prescribing and dispensing via the Internet.

